# Old concepts, new molecules and current approaches applied to the bacterial nucleotide signalling field

**DOI:** 10.1098/rstb.2015.0503

**Published:** 2016-11-05

**Authors:** Angelika Gründling, Vincent T. Lee

**Affiliations:** 1 Section of Microbiology and MRC Centre for Molecular Bacteriology and Infection, Imperial College London, London SW7 2AZ, UK; 2 Department of Cell Biology and Molecular Genetics, University of Maryland at College Park, College Park, MD 20742, USA

**Keywords:** bacteria, signalling nucleotide, receptor protein, effector protein, nucleotide detection, biosensor

## Abstract

Signalling nucleotides are key molecules that help bacteria to rapidly coordinate cellular pathways and adapt to changes in their environment. During the past 10 years, the nucleotide signalling field has seen much excitement, as several new signalling nucleotides have been discovered in both eukaryotic and bacterial cells. The fields have since advanced quickly, aided by the development of important tools such as the synthesis of modified nucleotides, which, combined with sensitive mass spectrometry methods, allowed for the rapid identification of specific receptor proteins along with other novel genome-wide screening methods. In this review, we describe the principle concepts of nucleotide signalling networks and summarize the recent work that led to the discovery of the novel signalling nucleotides. We also highlight current approaches applied to the research in the field as well as resources and methodological advances aiding in a rapid identification of nucleotide-specific receptor proteins.

This article is part of the themed issue ‘The new bacteriology’.

## 1. The principles of nucleotide signalling molecules and networks

**Figure 1.**
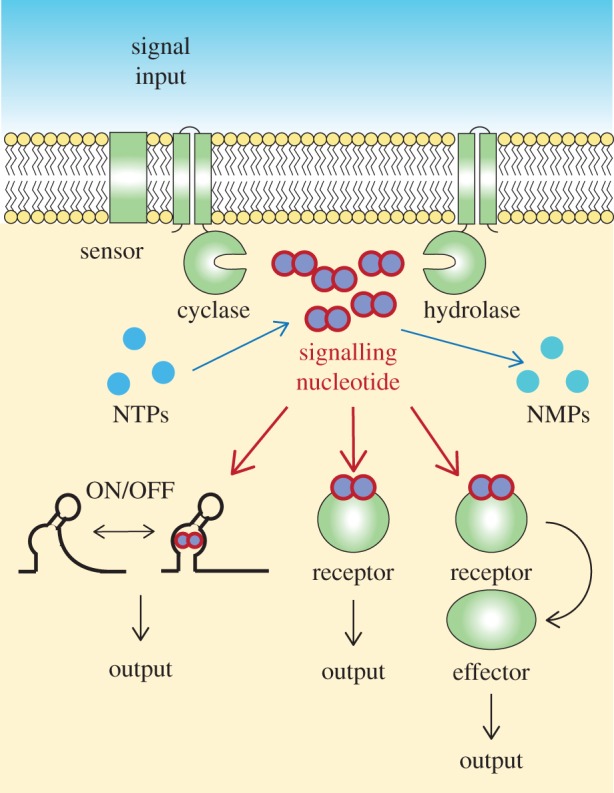
Schematic of nucleotide signalling pathway in bacteria. An input signal is sensed by a dedicated sensor protein or directly by the cyclase or hydrolase enzymes. This will result in their activating or inhibiting and as a consequence lead to a change in the intracellular signalling nucleotide concentration. At high signalling nucleotide levels, the molecule will bind (i) to nucleotide-specific riboswitches to affect the transcription or translation of downstream effector proteins or (ii) to specific receptor proteins and either directly alter their function or (iii) allow them to interact with specific downstream effector proteins. The final output of this will be the activation or repression of specific cellular pathways, which depending on the signalling nucleotide and to name a few examples can range from c-di-GMP controlling flagella, pili and expolysaccaride production, to the stringent response alarmones (p)ppGpp diverting resources away from active growth to amino acid synthesis in order to promote cell survival under starvation conditions.

Nucleotide signalling molecules play key roles in the control of cellular pathways in all domains of life. While we focus in this article on recent advances in the bacterial nucleotide signalling field, particularly on methodological innovations, the original concept of a nucleotide as signalling molecule originated in the 1950s from the investigation on the adrenaline receptor and its signal transduction network in eukaryotic cells [1–3]. Our current thinking of how signalling nucleotides function is still based on this original concept and putting it into the framework of bacterial cells can be formulated as follows: bacteria are constantly exposed to a changing environment, and in order to survive, cells must be able to detect these changes and rapidly transmit a signal to coordinate an appropriate cellular response; among other signals, changes in the levels of specific signalling nucleotides play an important role in this adaptation. As is discussed below, a number of different signalling nucleotides have now been uncovered; these are produced and degraded by dedicated enzymes, which in the case of cyclic nucleotides (which many signalling nucleotides are) are so-called cyclase and phosphodiesterase or hydrolase enzymes (figure 1). The cellular levels of each signalling nucleotide depend on the combined activity and net output of the enzymes responsible for their synthesis and degradation. Environmental changes and stimuli can be sensed directly by the nucleotide synthesizing or degrading enzymes and alters their activity or by dedicated sensory proteins, which transmit the signal to the respective cyclase and hydrolase enzymes to adjust their activities (figure 1). The changes in the cellular level of the signalling nucleotide are then perceived by so-called receptor proteins, which assume different conformations in the nucleotide-bound state and the unbound state (figure 1). The receptor proteins themselves can function as output or so-called effector proteins and have different activities in the nucleotide-bound and unbound form (figure 1). Alternatively, the receptor proteins can further transmit the signal by interacting with downstream effector proteins to alter their activity (figure 1). More detailed information on pathways controlled by diverse bacterial signalling molecules can be found in a number of recent reviews [4–11]. In place of binding to specific receptor proteins, there are now many examples where signalling molecules can also bind to specific RNA structures, called riboswitches, and in this manner affect the transcription or translation of a downstream gene (figure 1) [12–16]. As discussed in a later section, conformational changes in riboswitches and receptor proteins upon signalling molecule binding make them useful tools for the construction of biosensors allowing the detection of specific signalling nucleotides in living cells. A key characteristic of such a signal transduction network is that one signalling molecule can control and coordinate multiple cellular pathways, such as coordination of flagella versus pili motility by cyclic-di-guanosine monophosphate (c-di-GMP) or repression of ribosomal and tRNA synthesis genes and activation of amino acid transport and synthesis genes by the stringent response signalling nucleotides guanosine tetraphosphate (ppGpp) and guanosine pentaphosphate (pppGpp). A key in providing a better understanding of the function of a signalling nucleotide and the network it controls lies with the identification of the specific receptor and effector proteins. In the final section of this review, we discuss current approaches that have aided in the rapid identification of novel receptor proteins, often on a genome-wide level. It should, however, also be noted that the outlined concept is a somewhat simplified view of how a signalling nucleotide network functions. The ability of signalling nucleotides to function on a local level (right at the spot where they are synthesized) rather than at a cell-wide level is a concept that is discussed in more detail in another article in this issue [17].

## 2. Recent work leading to the discovery of new signalling nucleotides

**Figure 2.**
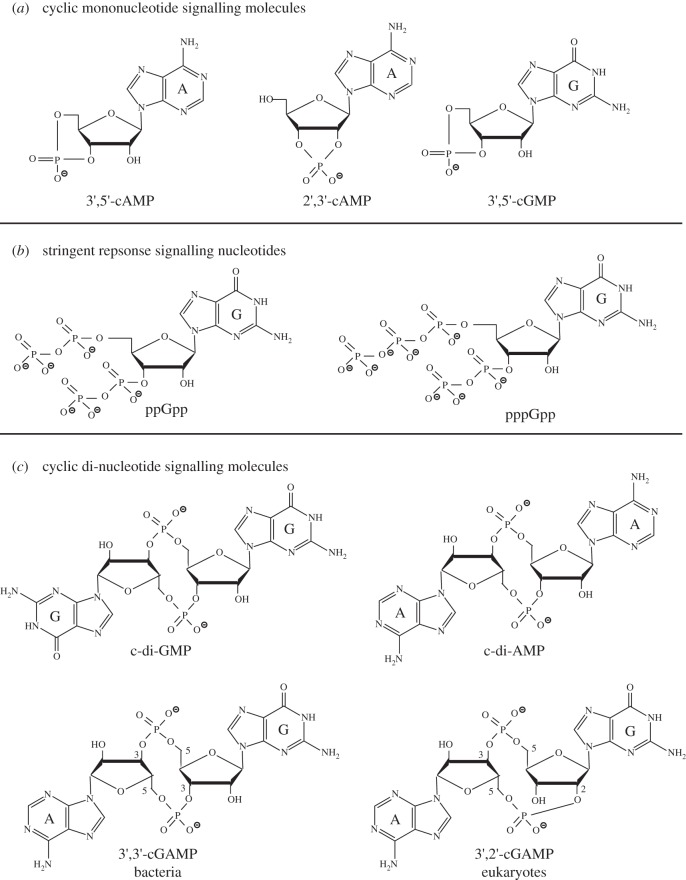
Chemical structures of (*a*) cyclic mononucleotide signalling molecules, (*b*) stringent response signalling nucleotides and (*c*) cyclic di-nucleotide signalling molecules.

More than a handful of different signalling nucleotides have now been detected in bacteria and their precise chemical structures elucidated (figure 2). Depending on the bacterial species, different signalling nucleotides are produced and it is clear that bacteria usually produce a multiplicity of them simultaneously. The starting building blocks of signalling nucleotides are usually the nucleotides ATP and GTP, and signalling molecules are often cyclic mononucleotides (figure 2*a*) or cyclic dinucleotides (figure 2*c*). The first signalling nucleotide identified in eukaryotic cells in 1958 and a few years afterwards in bacterial cells was the molecule cyclic adenosine monophosphate (cAMP; figure 2*a*) [2,3,18]. More specifically, the molecule identified at that time was 3′,5′-cAMP where the phosphate group is linked to hydroxyl groups attached to the 3′ and 5′ carbon residues within the ribose moiety of adenosine (figure 2*a*). Knowing the precise chemical structure of the nucleotide is important, as different nucleotide isoforms exist and these have different downstream effects and are produced and degraded by a different set of enzymes. Such differences in the production and effects between isoforms of signalling nucleotides have recently gained increased attention [19–21]. The second signalling nucleotide identified in bacteria in 1969 was a molecule originally referred to as ‘magic spot’, which was shown in 1970 to be a mixture of the two signalling nucleotides guanosine tetraphosphate (ppGpp) and guanosine pentaphosphate (pppGpp; figure 2*b*) [22–24]. These two nucleotides are often collectively referred to as stringent response signalling nucleotides, although recent work has indicated that ppGpp and pppGpp can have distinct functions and different effects on the regulation of cellular signalling processes [25]. In a recent report, experimental evidence for yet another version of a stringent response nucleotide, pGpp, was presented [26]. The bacterial nucleotide signalling field gained renewed interest during the 1990s shortly after the discovery of the cyclic dinucleotide c-di-GMP in 1987 (figure 2*c*) [27] (and see also reviews [9–11]). What attracted many researchers to this field at that point was the great complexity of this system (see review [28]). In contrast to the previously studied 3′,5′-cAMP and (p)ppGpp nucleotide systems, in which in the bacteria studied at that time only a few enzymes are responsible for their synthesis or degradation, a large number of proteins containing domains producing or degrading c-di-GMP were identified in a single bacterium, such as *Escherichia coli, Pseudomonas aeruginosa* or *Caulobacter crescentus* to name a few well-studied organisms (see also review [28]). The next novel signalling nucleotide, cyclic di-adenosine monophosphate (c-di-AMP), was discovered in 2008 (figure 2*c*) [29]. This was followed shortly afterwards by the identification of the cyclic AMP–GMP (cGAMP) hybrid molecules (figure 2*c*), first in bacteria in 2012 and subsequently in eukaryotic cells in 2013 [30–33]. Shortly after the identification of these hybrid cyclic di-nucleotide molecules, it was recognized that the bacterial and eukaryotic molecules are not identical but rather isoforms, with bacteria producing a 3′,3′-cGAMP and eukaryotic cells producing a 3′,2′-cGAMP molecule (figure 2*c*) [19–21]. Recent work has also confirmed that the signalling molecule 3′,5′-cyclic guanosine monophosphate (3′,5′-cGMP; figure 2*a*), long known to exist in eukaryotic cells, is also produced by bacteria such as *Rodospirillum centenum* and the plant pathogen *Xanthomonas campestris* [34,35]. Finally, several reports have described 2′,3′-cGMP and 2′,3′-cAMP isoforms of the classic 3′,5′ cyclic mononucleotides in eukaryotic cells (figure 2*a*) [36,37]. In addition, 2′,3′-cCMP and 2′,3′-cUMP nucleotides were also reported to be present in eukaryotic cells [38,39], and all these 2′,3′-cNMP are, at least in eukaryotic cells, thought to be produced during the RNA degradation process [40]. However, the exact functions of such 2′,3′-cNMP nucleotides and in particular if they also play a role as signalling molecules in bacterial cells have yet to be established. The discoveries of a number of novel nucleotide signalling molecules during the past 10 years has invigorated the field, attracted a large number of new researchers and sparked renewed interest in the two classic bacterial signalling nucleotides 3′,5′-cAMP and (p)ppGpp.

## 3. Methods for the detection and quantification of nucleotide signalling molecules

One important aspect in the field is the accurate detection and quantification of signalling nucleotides. For this, liquid chromatography coupled to mass spectrometry (LC–MS/MS) is currently the most widely used method [41,42]. In this approach, complete bacterial metabolite extracts are separated by LC, and the nucleotides of interest are detected based on their mass, and for a more accurate assignment, by their fragmentation pattern [42]. Signalling nucleotides within bacterial cells are often present in very small quantities, and hence the development of improved mass spectrometry equipment with better sensitivity increased the utility of this method for the bacterial nucleotide signalling field. Nucleotides with similar chromatographic behaviour, which can be isolated using the same metabolite extraction procedure, can be detected by this method simultaneously in a single run. An important issue for the detection and quantification of nucleotides in bacterial extracts is the use of an appropriate method for the preparation of the bacterial metabolite extracts. Some nucleotides are very labile and can be easily degraded during the preparation of the extracts or do not tolerate the heating step that often forms part of the extraction procedure. The concentration of a specific nucleotide within a bacterial extract can be quantified by comparing its signal intensity with that obtained from standards of known concentration and for the most accurate quantification extracts are spiked with a known concentration of a non-radioactive heavy isotope-labelled version of the signalling nucleotides one wishes to quantify. This labelled nucleotide will have the same chromatographic and ionization behaviour as the nucleotide to be quantified and serves therefore as an ideal internal calibrator to account for any ion suppression observed when analysing complex mixtures such as bacterial metabolite extracts [41–44]. While such internal isotope-labelled standards are extremely important for an accurate quantification of nucleotide levels, they are currently not commercially available and need to be synthesized by the user, most often using recombinant cyclase enzymes.

**Figure 3.**
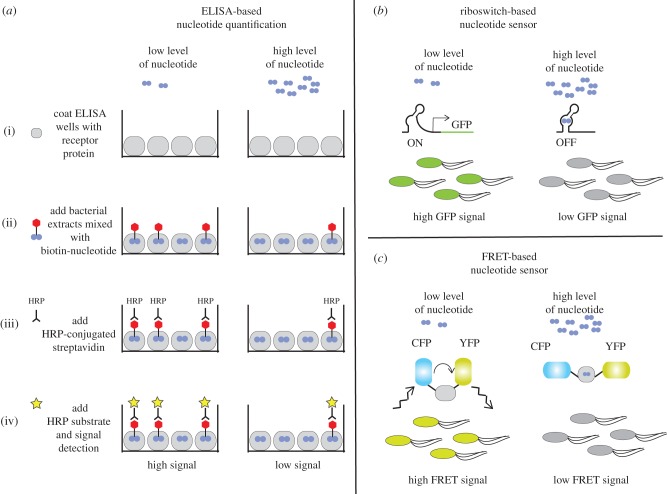
Methods for the detection of signalling nucleotides in bacterial extracts or within living cells. (*a*) Detection of nucleotides in cell extracts using an ELISA-based method. (*b*) Schematic of a riboswitch-based sensor for the detection of a specific signalling nucleotide in living cells. (*c*) Schematic of a FRET-based sensor for the detection of a signalling nucleotide in living cells.

The LC–MS/MS-based method is currently the most frequently used approach for the detection and quantification of signalling nucleotides in bacterial extracts, but it requires highly specialized equipment and expertise. A good alternative method that requires less specialized equipment and can therefore be more easily and routinely performed is an ELISA-based method. This method is frequently used for the detection of 3′,5′-cAMP in eukaryotic extracts using commercially available kits. A modified ELISA approach was recently described for the quantification of c-di-AMP in bacterial extracts [45]. For this approach, bacterial metabolite extracts are mixed with a known concentration of biotinylated-c-di-AMP and applied to a well of a 96-well plate that has been coated with a c-di-AMP-specific receptor protein (figure 3*a*). The amount of the biotinylated-c-di-AMP that binds to the receptor proteins depends on the concentration of the c-di-AMP in the extract (figure 3*a*). The amount of biotinylated-c-di-AMP retained in the well is subsequently quantified using, for instance, horseradish peroxidase-conjugated streptavidin and an appropriate signal detection kit. Based on the signal obtained compared with that of a simultaneously determined standard curve, the amount of c-di-AMP contained in bacterial extracts can be calculated (figure 3*a*). Performing an ELISA analysis is relatively inexpensive and fast, thus allowing the processing of multiple samples in a single run. However, usually only a single nucleotide is detected within an experiment and a careful calibration, and standard curve determination is required for each experiment.

The above-described methods are designed for the detection and quantification of signalling nucleotides within large populations of bacterial cells, and are often employed to measure differences in nucleotide levels after exposing bacteria to different environmental conditions or between wild-type and mutant bacteria. Methods that can report on nucleotide levels directly within living cells and on a single cell level have also been developed [46–49]. For instance, transcriptional fusion constructs have been created between nucleotide-responsive promoter elements and genes coding for fluorescent proteins [49]. Alternatively, nucleotide-specific riboswitches have been adapted for the construction of biosensors by coupling them to fluorescence probes or the expression of fluorescent proteins [47]. In the example depicted in figure 3*b*, at a low cellular nucleotide concentration, the riboswitch will be in the ON state, and bacteria will be highly fluorescent, but as the cellular nucleotide concentration increases, the riboswitch will switch to the nucleotide-bound OFF state, and bacteria will be non- or only dimly fluorescent (figure 3). Changes in fluorescent signal can be measured on a population level or in individual cells by fluorescence microscopy. A second type of biosensor is a fluorescence resonance energy transfer (FRET)-based sensor (figure 3*c*). For this sensor, a nucleotide-binding receptor protein is sandwiched between two fluorescent proteins with suitable excitation and emission wavelengths, such as a cyan fluorescent protein (CFP) and a yellow fluorescent protein (YFP). If the fluorescent proteins are in close enough proximity, one protein can be excited (in this case CFP) and the energy transferred to the second protein (in this case YFP) and emission by the second protein can be detected (figure 3*c*). In the example shown in figure 3*c*, a high FRET signal is observed in bacterial cells with a low level of signalling nucleotide. When the signalling nucleotide concentration in the cell increases, the nucleotide will bind to the sandwiched receptor protein leading to a conformational change in the receptor protein, placing the CFP and YFP proteins further apart and decreasing the FRET signal (figure 3*c*). The changes in FRET signal and fluorescence can again be measured on a population level or in individual cells by a fluorescence microscopy analysis. c-di-GMP-specific FRET sensors have been used to visualize changes in nucleotide levels in several Gram-negative bacteria [46]. For example, a c-di-GMP-specific-FRET sensor was used to detect the uneven distribution of c-di-GMP between the flagellated motile cell and the surface-attached stalk cell that are produced after asymmetric cell division of *Caulobacter crescentus*: the stalk cell retained a higher level of c-di-GMP than the motile daughter cell [46]. A similar asymmetric c-di-GMP content in daughter cells was observed following cell division in the Gram-negative bacterial pathogen *P. aeruginosa* [46]*.* It was subsequently shown that the asymmetric distribution of a c-di-GMP-specific phosphodiesterase enzyme is responsible for this [50]. Therefore, besides the ability to detect differences in nucleotide levels on a single cell level, such FRET-based biosensors make it possible to follow changes in nucleotide levels in real time, providing a unique view on the temporal aspects of nucleotide signalling processes.

## 4. Genome-wide approaches aiding in the identification of nucleotide receptor proteins

Discovering the receptors of signalling nucleotides is a critical aspect of understanding the molecular mechanism of regulation. The first bacterial receptor protein that was identified was a 3′,5′-cAMP binding protein identified in *E. coli* and called CAP or CRP. It was identified through two different biochemical fractionation approaches: (i) by restoration of a biochemical function and (ii) by binding to radiolabelled 3′,5′-cAMP. For the first approach, a mutant *E. coli* strain that produced cAMP but did not produce β-galactosidase in response to increased cAMP levels was used [51]. By purifying the activity from wild-type cells that stimulated the production of β-galactosidase in the mutant cell lysate, the catabolite-activating protein (CAP) was isolated [51]. The other approach identified the 3′,5′-cAMP receptor protein (referred to as CRP) by incubating protein fractions derived from a wild-type *E. coli* strain with radiolabelled cAMP and identifying proteins that co-precipitated in an ammonium sulfate precipitated step with the radiolabelled nucleotide [52]. Identification of CAP/CRP led to a major advance in our understanding of transcriptional regulation in bacterial cells. These early studies revealed the importance of the identification of receptor proteins of signalling nucleotides and highlighted the challenges and difficulties in identifying such receptors.

**Figure 4.**
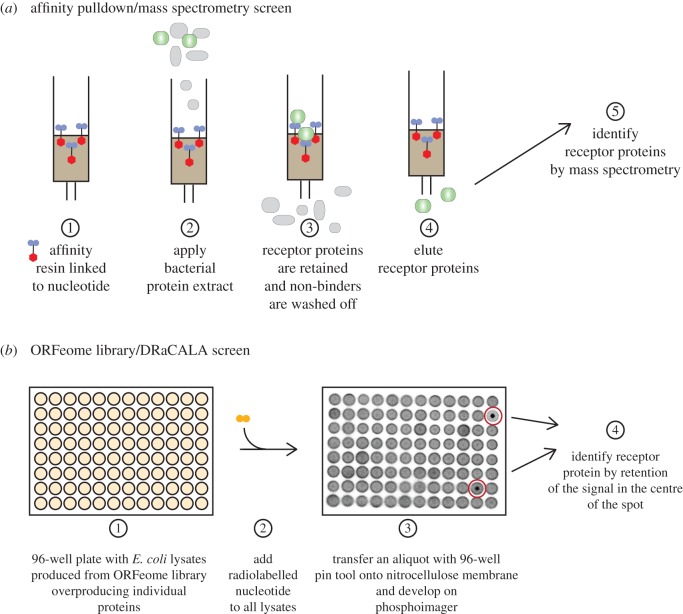
Schematic of genome-wide approaches for the identification of receptor proteins. (*a*) Affinity pull-down/mass spectrometry approach. Bacterial extracts are applied to a matrix coupled to a specific signalling nucleotide. Most bacterial proteins will pass through the column, whereas specific receptor proteins are retained on the column. Bound receptor proteins (or complexes) are eluted and subsequently identified by a mass spectrometry approach. (*b*) DRaCALA-based ORFeome library screen. *E. coli* lysate overproducing a specific ORFeom protein are arrayed out in 96-well plates. Lysates are mixed with a radiolabelled nucleotide and a small aliquot is subsequently spotted onto a nitrocellulose membrane. A positive interaction between an ORFeome protein and the signalling nucleotide is detected when the radioactive ligand remains bound to the protein in the centre of the spot, whereas in the case of non-interacting proteins, the radioactive ligand will diffuse outwards along the whole spot.

The initial characterization of a c-di-GMP receptor is also instructive in understanding the difficulty of receptor discovery for cyclic-di-nucleotides. The Benziman laboratory, which first characterized c-di-GMP as a signalling molecule that activates the bacterial cellulose synthase, proposed that a part of the cellulose synthase complex binds c-di-GMP [27]. Subsequent studies using UV-mediated photolabelling of radiolabelled c-di-GMP identified BcsB as the part of the cellulose synthase complex that binds c-di-GMP [53]. Later work however showed that it is actually a different protein of the complex, namely BcsA, that binds c-di-GMP via its PilZ domain [54]. Subsequent structural studies revealed that binding of the nucleotide to BcsA removes the PilZ domain from the catalytic site of the cellulose synthase [55,56]. The identification of the PilZ domain allowed a sequence-based bioinformatics approach to identify a number of other c-di-GMP receptors. However, PilZ domains were not able to explain all c-di-GMP-regulated phenotypes in the diverse set of organisms that used c-di-GMP signalling. In the past 10 years, several approaches have been employed to systematically identify c-di-GMP receptors, including UV cross-linking/mass spectrometry identification, affinity pull-down and mass spectrometry identification, and screening through open reading frame libraries (ORFeomes; figure 4).

One approach for the identification of receptor proteins is through direct UV cross-linking of radiolabelled nucleotides to such receptors followed by the identification of the cross-linked polypeptide by tandem mass spectrometry. This strategy has allowed the identification of additional c-di-GMP receptor proteins, highlighting that photocross-linking and subsequent protein identification is a feasible approach and can lead to the identification of new receptors [57]. The above-mentioned approach was improved using modified cyclic dinucleotides coupled to affinity resin, which allowed for the purification of receptor proteins followed by their identification through mass spectrometry (figure 4*a*). The cyclic dinucleotide can be coupled to a biotin tag or directly to the resin through activated groups [58–60]. In the search for c-di-AMP binding proteins, both types of affinity resins were used. Biotinylated c-di-AMP coupled to magnetic streptavidin–agarose beads allowed the identification of KtrA, a component of the potassium transporter, in *S. aureus* [59], whereas in the case of *Listeria monocytogenes*, coupling of c-di-AMP to epoxy-activated sepharose beads lead to the identification of the pyruvate carboxylase as a c-di-AMP binding protein, PgpH, a phosphodiesterase that linearizes c-di-AMP and several other receptor proteins [60,61]. A modified form of c-di-GMP with an extended 2′OH linker and a reactive cross-linker can interact with a diverse set of known binding proteins, indicating that this type of modification is well tolerated by macromolecular receptors [58]. Further development of molecules with other capturing technologies such as click or photoclick moieties may improve capture and identification of cyclic dinucleotide interacting proteins.

Another parallel approach for the identification of receptor proteins is to empirically test all open reading frames or proteins encoded within a bacterial genome for their ability to bind to a specific nucleotide. In this approach, each protein is heterologously expressed in *E. coli* and lysates are generated (figure 4*b*). The individual lysates are then tested for binding to the signalling nucleotide using the differential radial capillary action of ligand assay (DRaCALA; figure 4*b*) [62]. This genome-wide approach has allowed the identification of several new proteins that interact with cyclic dinucleotides. For example, a DRaCALA-based screen of the *S. aureus* ORFeome library for c-di-AMP and (p)ppGpp receptors identified PstA and KdpD as c-di-AMP receptors, confirmed KtrA as a receptor and identified several small GTPases as novel (p)ppGpp receptors [59,63]. A screen for c-di-GMP binding proteins from *E. coli* identified the GIL domain in BcsE as a receptor and confirmed the binding of c-di-GMP by a number of known receptors [64]. Two screens for binding proteins of c-di-GMP and pGpG were performed with a *Vibrio cholerae* ORFeome library. The c-di-GMP screen identified MshE as a receptor, which revealed that a new family of type II secretion system and type IV pili ATPases can bind c-di-GMP [65]. The pGpG screen revealed that the oligoribonuclease Orn binds pGpG [66] and serves as the primary phosphodiesterase B to break pGpG down into GMP [66,67]. These results suggest that the DRaCALA-based screening method can provide a genome-level perspective of nucleotide binding proteins.

Together, these studies demonstrate that a number of new approaches can identify receptors of nucleotide signalling molecules. The two main genome-wide approaches, affinity pull-down followed by mass spectrometric protein identification and DRaCALA-based ORFeome screening (figure 4), are complementary approaches that enhance discovery of these important receptors. Each approach has advantages; the use of nucleotide with functionalized cross-linkers is broadly applicable and can be used on any bacterium that may use cyclic dinucleotide signalling pathways. However, the ability to identify receptor proteins is limited by protein abundance and the sensitivity of mass spectrometry. Nonetheless, improvements in mass spectrometry technology should enhance the identification of new receptor proteins, using various affinity resin and capture compounds. The benefit of DRaCALA-based screens is that protein expression within the endogenous host does not depend on specific growth conditions. However, this approach requires the availability of an ORFeome library for the expression of the individual proteins in a heterologous host, that a single gene encodes the receptor protein and that heterologous expression of the protein is not toxic to *E. coli*. Future use of these two complementary methods will most certainly lead to the discovery of additional receptor proteins that bind cyclic dinucleotides and other signalling nucleotides, will enhance our understanding of secondary nucleotide signalling systems and likely reveal novel concepts and signalling pathways in bacteria.
